# Paroxysmal sympathetic hyperactivity concurrent with hypothalamic injury in a patient with intracerebral hemorrhage: A case report

**DOI:** 10.1097/MD.0000000000030058

**Published:** 2022-08-12

**Authors:** Sung Ho Jang, Kyu Hwan Choi

**Affiliations:** Department of Physical Medicine and Rehabilitation, College of Medicine, Yeungnam University, Daegu, Republic of Korea.

**Keywords:** diffusion tensor imaging, hypothalamus, intracerebral hemorrhage, intraventricular hemorrhage, paroxysmal sympathetic hyperactivity

## Abstract

**Background:**

Paroxysmal sympathetic hyperactivity (PSH) is characterized by exacerbated sympathetic discharge following severe brain injury. Here, we reports a patient diagnosed with PSH after ICH concurrent with hypothalamic injury, as demonstrated by diffusion tensor imaging (DTI).

**Methods:**

A 27-year-old man patient was diagnosed with spontaneous intraventricular hemorrhage and intracerebral hemorrhage in both frontal lobes. Two months after onset, brain magnetic resonance imaging of the brain revealed a leukomalactic lesion in the hypothalamus. Three months after the onset, he presented with intermittent high fever, tachycardia, tachypnea, systolic hypertension, diaphoresis, and aggravated rigidity. Infection was ruled out by a physical examination, laboratory tests, and radiological studies. After administrating morphine and bromocriptine, the clinical manifestations improved dramatically.

**Results:**

PSH after intracranial hemorrhage concurrent with the hypothalamic injury. Fractional anisotropy and mean diffusivity values of DTI were obtained in the hypothalamus. No significant difference in fractional anisotropy value was observed between the patient and control group (10 age-matched healthy male subjects) (*P* > .05). On the other hand, the mean diffusivity value was higher in the patient group than in the control group (*P* < .05), indicating hypothalamic injury.

**Conclusion:**

PSH concurrent with hypothalamic injury was observed in a patient with stroke. This study suggests that DTI can be a useful imaging method for evaluating the hypothalamic state of patients presenting with PSH after brain injury.

## 1. Introduction

Paroxysmal sympathetic hyperactivity (PSH) is characterized by cyclic and simultaneous appearance of signs and symptoms secondary to exacerbated sympathetic discharge following severe acquired brain injuries.^[1]^ A precise and prompt diagnosis of PSH is clinically important because it is a potentially life-threatening neurological emergency.^[1–3]^ In addition, it is associated with greater morbidity, longer hospitalization, and poorer outcomes.^[1–3]^ Many studies have suggested diagnostic criteria based on the clinical features with various diagnostic terminologies (e.g., sympathetic storms or dysautonomia paroxysm).^[3–6]^ Recently, a consensus group suggested the paroxysmal sympathetic hyperactivity assessment measure to reduce diagnostic ambiguity. This measure is a diagnostic criterion that uses a probabilistic diagnostic system consisting of the Clinical Feature Scale that categorizes the severity of the sympathetic signs during each episode, and a diagnostic likelihood tool that gauges the certainty of the diagnosis of PSH based on the presence of characteristic features of the disorder in 2014.^[3,7]^

The pathophysiological mechanisms underlying PSH have not yet been elucidated. On the other hand, 2 main hypotheses have been suggested: (1) disconnection theory—a loss of normal autonomic regulatory control mechanisms-simple disconnection between the cortical inhibitory centers (the insula and cingulate cortex) and the sympathetic centers (hypothalamus, diencephalon, and upper brainstem); and (2) the excitatory/inhibitory ratio model—paroxysms are driven by the abnormal processing of afferent stimuli within the spinal cord following disconnection of descending inhibitory pathways.^[1,6,8]^

The hypothalamus is one of the most important centers regulating the autonomic nervous system of the human brain, and hypothalamic injury results in autonomic dysfunction, including sympathetic overflow.^[6,8]^ On the other hand, a precise estimation of the hypothalamus in the live human brain is limited because of the anatomical characteristics of the hypothalamus: very small and deep location within the brain white matter.^[9,10]^ By contrast, diffusion tensor imaging (DTI) has enabled the evaluation of the hypothalamus in a live human brain.^[11–13]^ Several studies using DTI have reported hypothalamic injuries in patients with various brain diseases, including traumatic brain injury, hypoxic-ischemic brain injury, and multiple sclerosis.^[12–17]^ On the other hand, no DTI study on the hypothalamic injury in patients with PSH has been reported.

This paper reports a patient diagnosed with PSH after an intracerebral hemorrhage concurrent with a hypothalamic injury demonstrated on DTI.

## 2. Case report

**Figure 1.**
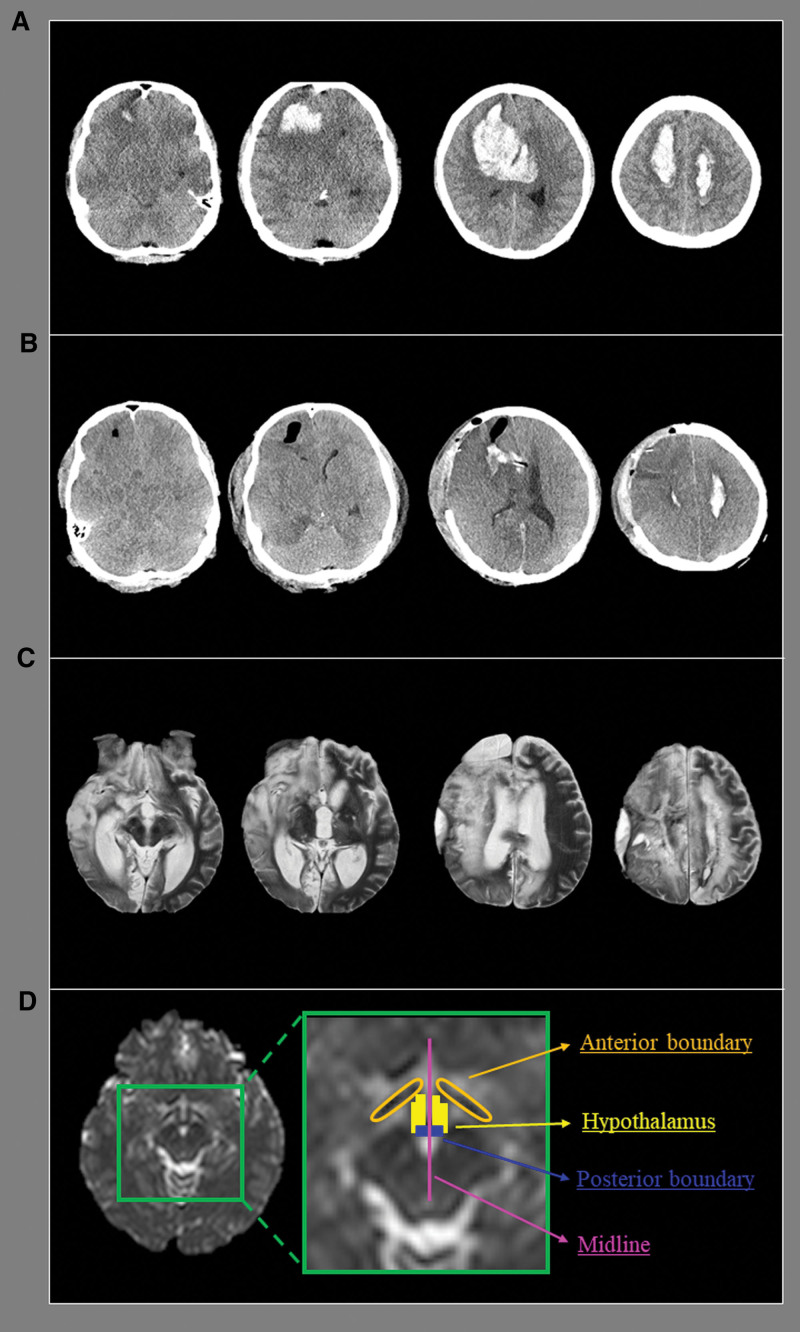
(A) Brain computed tomography images at the stroke onset show a spontaneous intraventricular hemorrhage and intracerebral hemorrhage. (B) Brain computed tomography images taken after the craniectomy and hematomal removal operation show aggravated brain edema. (C) Brain magnetic resonance images at 2 months after onset reveal leukomalatic lesions in both fronto-parieto-occipital cortices, right temporal lobe, and hypothalamus. (D) Regions of interest for the hypothalamus are localized using the optic tract (anterior boundary), the mammillary body (posterior boundary), and the midline (medial boundary) at the level of the upper midbrain in the patient.

A 27-year-old male patient and 10 age-matched healthy male subjects (mean age 26.60 ± 3.86 years [22–35]) with no history of neurologic/psychiatric disease were recruited. The patient was diagnosed with spontaneous intraventricular hemorrhage and intracerebral hemorrhage in both frontal lobes at the neurosurgery department of a university hospital (Fig. 1A). The patient presented with severely impaired consciousness with a Glasgow Coma Scale score of 3 and a Coma Recovery Scale-Revised score of 0. The patient underwent craniectomy and hematoma removal. Subsequently, the patient underwent right frontotemporal lobectomy for brain swelling (Fig. 1B). Approximately 2 months after onset, he was transferred to the rehabilitation department of another university hospital. He was in a vegetative state with a Glasgow Coma Scale score of 6 and a Coma Recovery Scale-Revised score of 7. His vital signs (body temperature, respiration rate, heart rate, and blood pressure) were stable upon hospital admission. Brain magnetic resonance imaging showed leukomalatic lesions in both fronto-parieto-occipital cortices, right temporal lobe, and hypothalamus (Fig. 1C).

Comprehensive rehabilitation therapy was administered after hospital admission. Approximately 3 months after onset, he suddenly presented with intermittent high fever (max: 39.1°C, 37.7 to 38.8°C), tachycardia (114–130 beats per minute), tachypnea (>23 breaths/min), and systolic hypertension (>140 mm Hg). He also showed severe diaphoresis and aggravated rigidity in all 4 extremities. Blood tests revealed a mildly elevated white blood cell count (11,750 cells/μL; normal range: 4000–10,000 cells), whereas other results were within the normal range: neutrophil differential count (67.3%; normal range: 40–74%), C-reactive protein level (0.538 mg/dL; normal range: 0.3–1 mg/dL), blood sugar level (101 mg/dL; normal range: 70–110 mg/dL), partial pressure of oxygen (87.8 mm Hg; normal range: 83–108 mm Hg), and procalcitonin level (0.094 ng/mL; normal range: 0–5 ng/mL), which are inflammatory biomarkers used to distinguish a bacterial infection from a nonbacterial infection with high sensitivity and specificity.^[18]^ The plain chest radiography did not reveal any definite evidence of pneumonia. In addition, he did not present with any signs of infection on physical examination, and no epileptiform discharge was observed on electroencephalography. Intravenous antipyretics and empirical antibiotics were administered to subside the high fever, considering the medical history of recurrent pneumonia during the early stages after onset. In contrast, high fever and other clinical manifestations, including tachypnea, tachycardia, hypertension, diaphoresis, and aggravated rigidity, did not subside. PSH was suspected based on clinical features. Therefore, appropriate medications were administered to the patient for PSH (bromocriptine 7.5 mg/day and intravenous morphine sulfate 5 g). After administering intravenous morphine, the fever, tachycardia, tachypnea, and hypertension normalized. Other clinical features, including diaphoresis and rigidity, were also improved. The paroxysmal sympathetic hyperactivity assessment measure score of the patient was 21 points (clinical feature scale: 13 [severe] and diagnostic likelihood tool: 8 of 11), indicating a probable diagnostic likelihood of PSH.^[7]^

This case report provides informed consent from patients and control subjects and was approved by the institutional review board of a university hospital.

### 2.1. Diffusion tensor imaging

The DTI data were acquired approximately 2 months after onset using a 6-channel head coil on a 1.5 T Philips GyroscanIntera (Philips, Best, Netherlands) with single-shot echo-planar imaging. For each of the 32 noncollinear diffusion-sensitizing gradients, 67 contiguous slices were acquired parallel to the anterior commissure-posterior commissure line. The imaging parameters were as follows: acquisition matrix = 96 × 96, reconstructed to a 192 × 192 matrix, field of view = 240 mm × 240 mm, TR = 10,398 ms, TE = 72 ms, parallel imaging reduction factor (SENSE factor) = 2, EPI factor = 59 and b = 1000 s/mm^2^, NEX = 1, slice gap = 0, and slice thickness = 2.5 mm. The eddy current-induced image distortions were removed using the affine multiscale 2-dimensional registration provided in the Oxford Centre for Functional MRI of the Brain Software Library. DTI-Studio software (CMRM, Johns Hopkins Medical Institute, Baltimore, MD) was used to evaluate the hypothalamus, which was identified by locating the anterior boundary of the optic tract and posterior boundary of the mammillary body at the level of the upper midbrain.^[19]^ Fractional anisotropy (FA) and mean diffusivity (MD) estimates were obtained for the hypothalamus. The regions of interest for the hypothalamus were localized using optic tract (anterior boundary), mammillary body (posterior boundary), and midline (medial boundary) at the level of the upper midbrain in the patient and control subjects (Fig. 1D).

### 2.2. Statistical analysis

Statistical analyses were performed using the SPSS software (version 25.0; SPSS, Chicago, IL). The analysis was conducted using Bayesian statistics to determine the differences in the FA and MD of the patients and the respective mean values of the control group.^[20]^ No significant difference in FA values was observed between the patient and control groups (*P* > .05). In contrast, the MD value was significantly higher in the patient group than in the control group (*P* < .05) (Table 1).

**Table 1:** Comparisons of diffusion tensor imaging parameters of the hypothalamus between the patient and control group (N = 10).

		Diffusion tensor imaging parameters	
		Patient	Controls
[Significance]*	FA	0.20	0.24 ± 0.02
		[0.06]	
	MD	2.08	1.04 ± 0.10
		[0.00]‡	
Estimated effect size† (95% CI)	FA	–2.033	
		(–4.160, 0.094)	
	MD	3.516	
		(2.733, 4.301)	

## 3. Discussion

The clinical features of PSH include paroxysmal fever, hypertension, tachycardia, tachypnea, diaphoresis, and abnormal posturing or dystonic movements.^[1–3]^ A diagnosis of PSH is commonly diagnosed based on typical clinical features because definite diagnostic criteria have not been established.^[3–6]^ This patient was diagnosed with PSH for the following reasons. First, according to the diagnostic criteria suggested by the consensus group in 2014, the clinical features of this patient-matched PSH were fever, systolic hypertension, tachycardia, tachypnea, diaphoresis, and aggravated rigidity, with a paroxysmal sympathetic hyperactivity assessment measure score of 21 points.^[7]^ Second, the possibility of other differential diagnoses, such as infection, hypoxia, seizure, or hypoglycemia, was excluded through various blood tests and electroencephalography. Third, the clinical features of PSH resolved rapidly with the administration of drugs for PSH (morphine and bromocriptine), especially morphine, which attenuates sympathetic overflow.^[6]^

DTI parameters of the hypothalamus in this patient were measured, and a high MD value compared to 10 age- and sex-matched healthy control subjects. The FA value represents the state of white matter organization by indicating the degree of directionality, whereas the MD value indicates the magnitude of water diffusion.^[21]^ Therefore, the increased MD value of the hypothalamus in this patient compared with that in the control subjects indicates injury to the hypothalamus. The hypothalamic injury in this patient was attributed to compression of the hypothalamus by severe brain edema after intracranial hemorrhage.

The hypothalamus is an important autonomic center of the central autonomic network. In particular, the paraventricular nucleus in the hypothalamus controls sympathetic tone.^[15,22,23]^ As a result, the hypothalamic injury can be accompanied by uncontrolled sympathetic symptoms, such as tachycardia, hypertension, and hyperthermia.^[12–14]^ Thus, the PSH of this patient was attributed at least partly to the hypothalamic injury. Therefore, an evaluation of the hypothalamus using DTI would be helpful for a precise diagnosis of PSH when a patient presents with clinical features indicating PSH, such as tachycardia, hypertension, and hyperthermia. Since the introduction of DTI, several studies have demonstrated hypothalamic injuries using DTI.^[11–13]^ Nevertheless, to the best of the authors’ knowledge, this is the first case study demonstrating PSH concurrent with the hypothalamic injury. However, this study was limited because it was based on a case report. Therefore, further studies involving larger numbers of subjects are required.

In conclusion, PSH concurrent with hypothalamic injury on DTI was demonstrated in a patient with stroke. This study suggests that DTI can be a useful imaging method for evaluating the hypothalamic state of patients presenting with PSH after brain injury.

## Author contributions

Sung Ho Jang: Study concept and design, Manuscript development and writing, Kyu Hwan Choi: Acquisition and analysis of data, Study concept and design, Acquisition and analysis of data, Manuscript authorization.

Conceptualization: Sung Ho Jang.

Data curation: Kyu Hwan Choi.

Methodology: Kyu Hwan Choi.

Writing—original draft: Sung Ho Jang.

Writing—review and editing: Kyu Hwan Choi.

## Footnotes

* [Significance]: a Bayesian hypothesis 1-tailed test for the probability that a member of the control population has a lower FA or higher MD score than the patient.

† Effective size for the difference between the patient and group of control subjects.

‡*P* < .05.
